# Localization Pattern of Dispatched Homolog 2 (DISP2) in the Central and Enteric Nervous System

**DOI:** 10.1007/s12031-023-02129-8

**Published:** 2023-06-27

**Authors:** Marvin Heimke, Florian Richter, Tillmann Heinze, Madlen Kunke, Thilo Wedel, Martina Böttner, Jan-Hendrik Egberts, Ralph Lucius, François Cossais

**Affiliations:** 1 Institute of Anatomy, Kiel University, Olshausenstrasse 40, 24098 Kiel, Germany; 2 Department of General, Thoracic, Transplantation and Pediatric Surgery, University Hospital Schleswig-Holstein, Kiel University, Kiel, Germany; 3 Department of Surgery, Israelite Hospital, Hamburg, Germany

**Keywords:** Dispatched homolog, DISP2, Enteric nervous system, Central nervous system, Aging

## Abstract

Dispatched homolog (DISP) proteins have been implicated in the regulation of hedgehog signaling during embryologic development. Although DISP2 has recently been associated with neuronal development and control of cognitive functions, its localization pattern in the mammalian central and peripheral nervous system has not yet been investigated. In this study, the Disp2 expression profile was assessed in human tissues from publicly available transcriptomic datasets. The DISP2 localization pattern was further characterized in the human and rat central nervous system (CNS), as well as within the colonic enteric nervous system (ENS) using dual-label immunohistochemistry. Colocalization of DISP2 with neuronal and glial markers was additionally analyzed in murine primary ENS culture. At transcriptomic level, DISP2 expression was predominant in neuronal cell types of the CNS and ENS. DISP2 immunoreactivity was mainly located within PGP9.5-positive neurons rather than in S100-positive glial cells throughout the nervous system. Investigation of human and rat brain tissues, colonic specimens, and murine ENS primary cultures revealed that DISP2 was located in neuronal cell somata, as well as along neuronal processes both in the human and murine CNS and ENS. Our results indicate that DISP2 is prominently localized within neuronal cells of the CNS and ENS and support putative functions of DISP2 in these tissues.

**Supplementary Information:**

The online version contains supplementary material available at 10.1007/s12031-023-02129-8.

## Introduction

Dispatched proteins are transmembrane proteins regulating the release of lipidated Hedgehog (Hh) proteins at the plasma membrane (Briscoe and Thérond 2013). The Dispatched protein was first described in 1999 by Burke et al. in *Drosophila* (Burke et al. 1999). So far, three members of the Dispatched homolog (DISP) protein family have been described in vertebrates: DISP1 (also known as DISPA), DISP2 (DISPB), and DISP3 (Katoh and Katoh 2005). In particular, DISP1 was shown to act synergistically with the membrane-associated Signal peptide-CUB-EGF domain-containing protein 2 (SCUBE2) to regulate long-range Hh signaling during development, including Sonic hedgehog (Shh) release (Cohen 2010; Tukachinsky et al. 2012). It is assumed that DISP transfers the cholesterol-tethered Shh to SCUBE2 at the plasma membrane (Briscoe and Thérond 2013). However, little is known about the localization and functions of other DISP members.

This is particularly the case for DISP2, which has recently been associated with the regulation of cognitive functions in genome-wide associations studies (GWAS). Indeed, DISP2-associated risk loci have been related to altered morphology of the transverse temporal gyrus (Cai et al. 2014), as well as to impaired cognitive functions and educational attainment (Davies et al. 2018; Lee et al. 2018; Savage et al. 2018) as determined using PheWas analysis (Buniello et al. 2019; Watanabe et al. 2019).

Although DISP2 localization has only been reported in islets of Langerhans within the human and mouse pancreas so far (Hald et al. 2012), a recent bioinformatics study based on single-cell transcriptomic expression datasets identified *DISP2* as a potential specific neuronal marker in the human and mouse brain (McKenzie et al. 2018). Furthermore, Nakano et al. demonstrated that *Disp2* is mainly expressed in the central nervous system (CNS) in zebrafish and *Disp2* mRNA accumulates specifically in cells of the telencephalon and ventral hindbrain, as well as in a discrete patch of cells in the gut in these animals (Nakano et al. 2004). However, a detailed analysis of the DISP2 localization pattern in neural tissues in mammals, including human, has not yet been performed.

In this study, we provide a DISP2 expression database analysis and an immunohistochemical characterization of DISP2 staining pattern in human and murine neural tissues, indicating that DISP2 is expressed in mammalian central and enteric neurons.

## Material and Methods

### Primary Culture of Enteric Nervous System

The primary culture of enteric nervous system (ENS) was obtained as previously described (Kneusels et al. 2021). In brief, dissected guts from e12.5 to e14.5 mouse embryos were minced mechanically and digested with 0.1% trypsin (Sigma-Aldrich) for 15 min at 37 °C. Cells were then treated with 0.01% DNase I (Sigma-Aldrich) for 15 min at 37 °C. Reaction was stopped by addition of DMEM/HAM’s F12 (1:1) medium supplemented with 10% v/v fetal calf serum (FCS, Pan-Biotech). Cells were seeded at a density of 4 × 10^5^ cells per well on 24-well plates (Cell + , Sarstedt). After 24 h, the medium was replaced by FCS-free DMEM/HAM´s F12 (1:1) supplemented with N2 (Pan Biotech), and cells were further grown for 48 h before fixation with 4% paraformaldehyde for 1 h.

### Tissue Preparation

For analysis of the human ENS, colonic specimens of patients, who underwent partial colectomy for non-obstructive colorectal carcinoma, were used as previously described (Cossais et al. 2021). Full-thickness specimens were harvested from the sigmoid colon at a safe distance (> 5 cm) from the tumor. Human brain specimens were retrieved post-mortem from five body donors (age range 74–88 years, 4 males and 1 female, supplementary table 1), who were recruited from the body donation program of the Institute of Anatomy, Kiel University, Germany. Brain tissue specimens were retrieved within 24 h post-mortem. Donors and patients gave a written consent to the use of their tissues for research purposes. Specimen analysis and collection have been approved by the local ethics committee of the Faculty of Medicine, Kiel University, Germany (B299/07). Rat brain and colonic tissues were obtained from Sprague–Dawley adult animals. Experiments were performed in agreement with the local Ethics Committee (V242-70,056/2015(91–7/15)) and in accordance with the 3R principles (Replacement, Reduction and Refinement) to reduce the number of animals sacrificed at our institute. Rat and human brain and colonic tissues were fixed (4% paraformaldehyde) for 24–48 h, dehydrated, embedded into paraffin wax, cut in sections (6 µm thickness), and subjected to immunohistochemistry (IHC). Rat colonic tissues were processed for whole-mount preparation as previously described (Wedel et al. 1998). The circular muscle layer was removed with micro-instruments under stereomicroscopic control, leaving the myenteric plexus attached to the thin longitudinal muscle layer for subsequent immunostaining.

### Immunohistochemistry

After rehydration tissue sections were pre-treated with citrate buffer (pH 6.0) in a microwave oven for 2 min at 800 W followed by 14 min at 140 W. After repeated washing with PBS, tissue sections or primary cell cultures were incubated overnight with following primary antibodies: mouse anti-DISP2 (F66A4B1, Developmental Studies Hybridoma Bank, 1:200), rabbit anti-PGP9.5 (318A, Cell Marque, 1:200), rabbit anti-S100 (Z-0311, DakoCytomation, 1:5000), goat anti-ChAT (AB144P, Millipore, 1:100), and rabbit anti-nNOS (160,870, Cayman Chemicals, 1:2000) in antibody diluent (Invitrogen). For immunofluorescence-staining, secondary antibodies, including anti-mouse AlexaFluor555 and anti-rabbit Alexafluor488 (Invitrogen), were diluted in antibody diluent (Invitrogen) and incubated for one hour at room temperature. Nuclei were counterstained with Hoechst 33342 (Sigma-Aldrich). For double-labelled immunohistochemistry, Brightvision one step detection system anti-mouse AP and anti-rabbit HRP were used (ImmunoLogic). Immpact Vector red and Immpact DAB EqV were used as substrates for AP and HRP, respectively (Vector Laboratories). Nuclei were counterstained with hematoxylin. Blank controls were performed for all tissue types by omitting primary antibodies (supplementary Fig. 1–5). Image acquisition was performed on a digital fluorescence inverted microscope (Keyence BZ-X800) using the software Keyence BZ-X800 Viewer and Keyence BZ-X800 Analyzer Version 1.1.1.8 (Keyence Corporation). Confocal microscopy was performed with a “Facility Line” system (Abberior) based on an IX-83 inverted microscope (Olympus), running the Imspector 16.3.11308 software. Image composition was performed with Adobe Photoshop Version 22.3.0.

### Expression Database Analysis

Normalized gene expression values (nTPM) for *DISP2* mRNA across human tissues and brain cell types were obtained from the Human Protein Atlas (version 22.0; https://www.proteinatlas.org/about/download; RNA GTEx tissue gene data, RNA single cell type data) (Sjöstedt et al. 2020; Karlsson et al. 2021). Single-cell expression data for the human enteric nervous system were obtained from a previously published dataset (Fawkner-Corbett et al. 2021) and analyzed using the software R (version 4.1.0) running the Seurat library (version 4.0.3) (Hao et al. 2021).

## Results

**Fig. 1:**
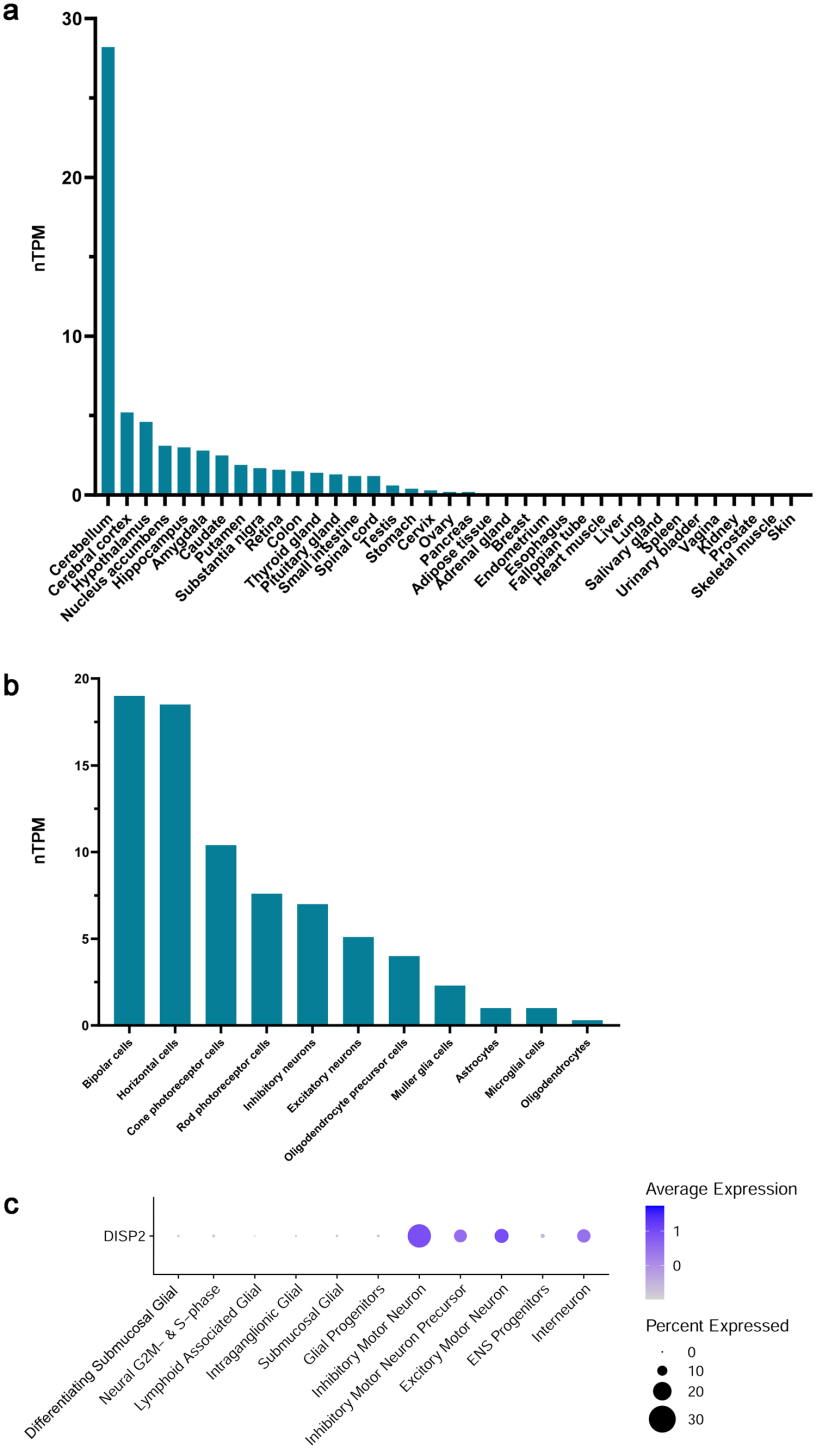
DISP2 expression pattern in human tissues and neuronal cell types. Normalized expression as transcripts per million (nTPM) for *DISP*2 in different human tissues as obtained from GTEx/Human Protein Atlas (**a**). Normalized expression for DISP2 in brain-associated cell types as obtained from the Human Protein Atlas (**b**). Dot-plot of average expression profile for DISP2 in single cells of the human enteric nervous system as obtained from Fawkner-Corbett et al. (2021) (**c**)

Expression profiles of *DISP2* were assessed in human organs using available databases (GTEx, Human Protein Atlas). *DISP2* was expressed at the highest level in the human brain, as well as in colonic tissues (Fig. 1a, nTPM > 1.5). Assessment of additional single-cell expression datasets of human brain tissues obtained from the Human Protein Atlas indicates that *DISP2* is expressed at higher levels in neuronal than in glial cell types (Fig. 1b). To determine whether *DISP2* may similarly be expressed in neuronal cells outside of the CNS, the expression profile of *DISP2* was analyzed in one single-cell transcriptomic dataset for the human ENS (Fawkner-Corbett et al. 2021). DISP2 was expressed at higher levels in all enteric neuronal cell types than in enteric glial cell populations (Fig. 1c). Additionally, *DISP2 *was expressed in neuroendocrine cells (data not shown).

**Fig. 2:**
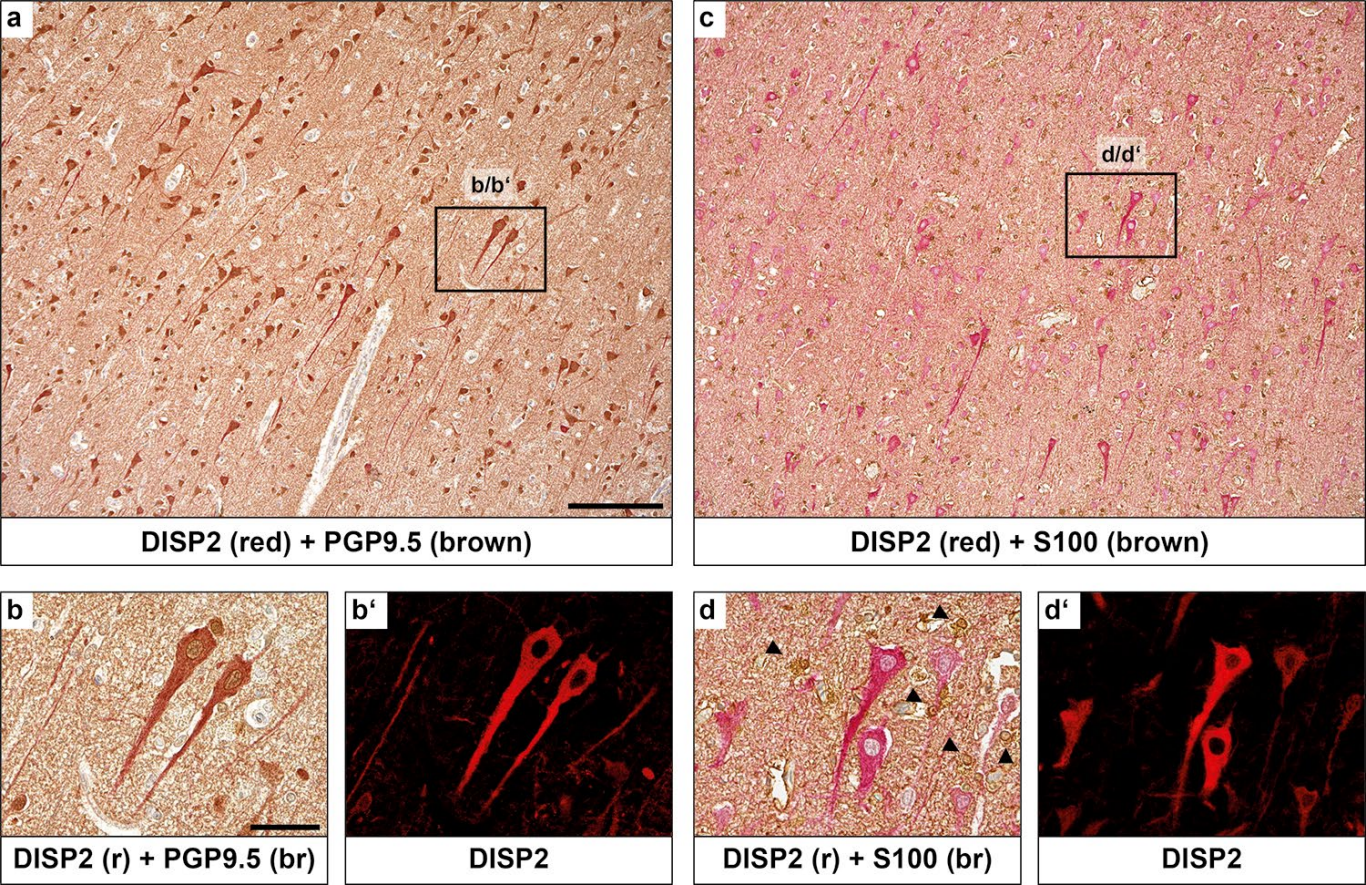
Cell-specific localization of DISP2 in the adult human brain. Immunohistochemical staining for DISP2 (red, **a**, **b**, **c**, **d**) and the neuronal marker PGP9.5 (brown, **a**, **b**) or the glial marker S100 (brown, **c**, **d**). Nuclei were counterstained with hematoxylin (blue). Costaining of DISP2 with PGP9.5 shows colocalization in neuronal somata and processes (**b**). Immunofluorescence microscopy of the same section (**b**′) for DISP2 (red) confirmed colocalization of DISP2 with PGP9.5, excluding the cell nuclei. Costaining of DISP2 with the glial marker S100 on consecutive sections (**d**) reveals no colocalization of DISP2 with S100-positive glial cells (marked by arrowheads), confirmed by immunofluorescence microscopy of the same section (**d**′) (scale bar in a = 200 µm, b = 100 µm; *n* = 5)

The DISP2 localization pattern in the CNS was further investigated by immunohistochemistry of human cortical brain sections. DISP2 immunoreactivity was observed in all PGP9.5-positive neurons and was particularly prominent in pyramidal neurons (Fig. 2a, b). Fluorescence microscopy for DISP2 on same sections (b′) confirmed the localization of DISP2 in PGP9.5-positive neuronal cell somata and processes, excluding the cell nuclei. Costaining of DISP2 with the glial marker S100 on consecutive sections (c, d) revealed no colocalization of DISP2 with S100-positive glial cells, as confirmed by fluorescence microscopy (d′).

**Fig. 3:**
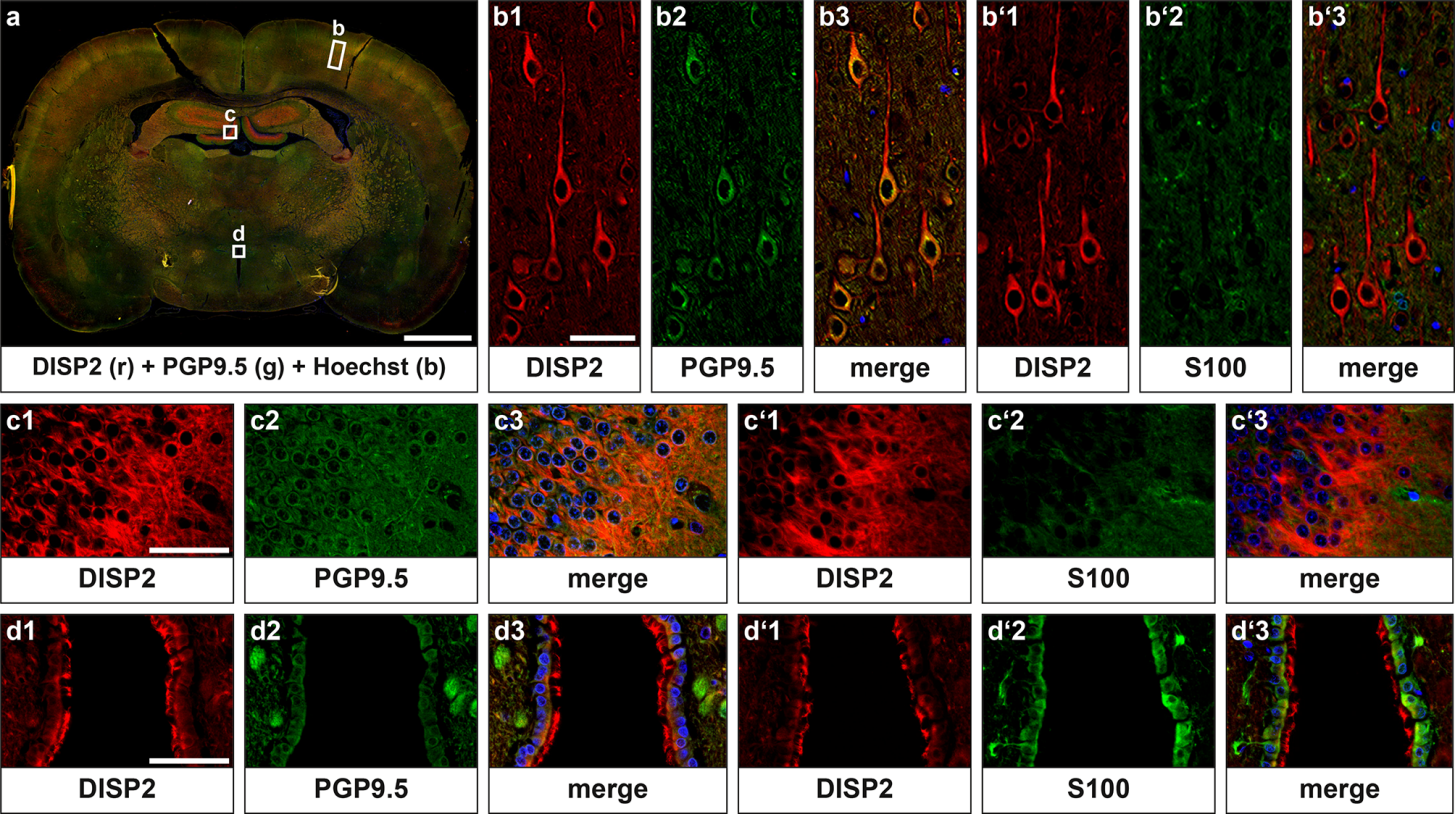
DISP2 localization pattern in the adult rat central nervous system. Immunohistochemical staining for DISP2 was performed on frontal sections of adult rat brain. Magnifications for the motor cortex (**b**, **b**′) and the dentate gyrus of the hippocampus (**c**, **c**′) show accumulation of DISP2 (red, **b**1, **c**1) in PGP9.5- (green, **b**2, **c**2) positive neuronal cell somata and processes. Costaining with the glial marker S100 (green, **b**′2, **c**′2, **d**′2) on consecutive sections revealed no colocalization with DISP2 but strong DISP2 immunoreactivity at the apical pole of S100-positive ependymal cells (**d**). Nuclei were counterstained with Hoechst (blue), and corresponding merge images are shown (**b**3, **b**′3, **c**3, **c**′3, **d**3, **d**′3) (scale bar in **a** = 2000 µm, **b**, **c**, **d** = 50 µm; *n* ≥ 3)

In adult rats, DISP2 staining was observed in PGP9.5-positive neurons throughout the brain (Fig. 3), but not in S100-positive glial cells (Fig. 3b′, c′). More particularly, DISP2 staining was detected in neuronal cell somata and processes of cortical (Fig. 3b) and hippocampal neurons (Fig. 3c). No DISP2 immunoreactivity was observed in neuronal nuclei. Of note, DISP2 signals were also detected at the apical pole of S100-positive ependymal cells surrounding the ventricles (Fig. 3d, d′), while the epithelial cells of the choroid plexus exhibited no DISP2 immunoreactivity (data not shown).

**Fig. 4:**
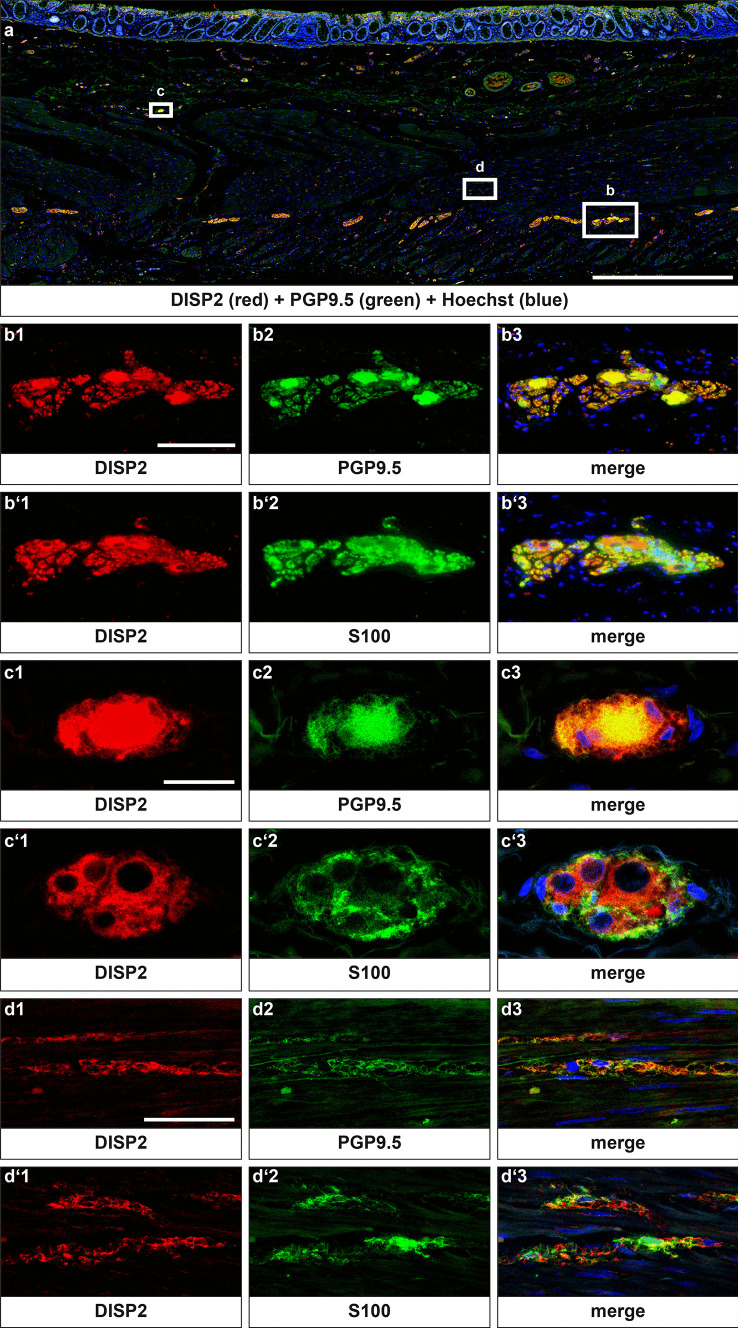
Site-specific localization of DISP2 in the adult human colon. Overview of a full-thickness colonic cross-section showing immunoreactivity for DISP2 area-wide throughout enteric ganglia (**a**). Higher magnification of a myenteric (**b**/**b**′) and submucosal ganglion (**c**/**c**′) shows colocalization of DISP2 (red, **b**1, **c**1) with the neuronal marker PGP9.5 (green, **b**2, **c**2) predominantly in the cell somata but excluded in the cell nuclei. In the circular musculature, distinct DISP2 signals were detectable in PGP9.5-positive nerve fiber strands (**d**). Costaining with the glial marker S100 (green, **b**′2, **c**′2, **d**′2) was performed on consecutive sections showing only limited colocalization with DISP2. Nuclei were counterstained with Hoechst (blue), and corresponding merge images are indicated (**b**3, **b**′3, **c**3, **c**′3, **d**3, **d**′3) (scale bar in a = 500 µm, b = 100 µm; *n* = 3)

DISP2 localization pattern within intestinal tissues was investigated using cross-sections of the human sigmoid colon (Fig. 4). DISP2 immunoreactivity was detected in PGP9.5-positive neuronal somata, and to smaller extent in ganglionic neuropil in both, the myenteric (Fig. 4b) and the submucosal (Fig. 4c) plexuses, while colocalization with S100 was limited. Nerve fiber strands along the circular musculature showed clear DISP2 immunoreactivity (Fig. 4d). PGP9.5-positive neuronal cell nuclei showed no DISP2 signals.

**Fig. 5:**
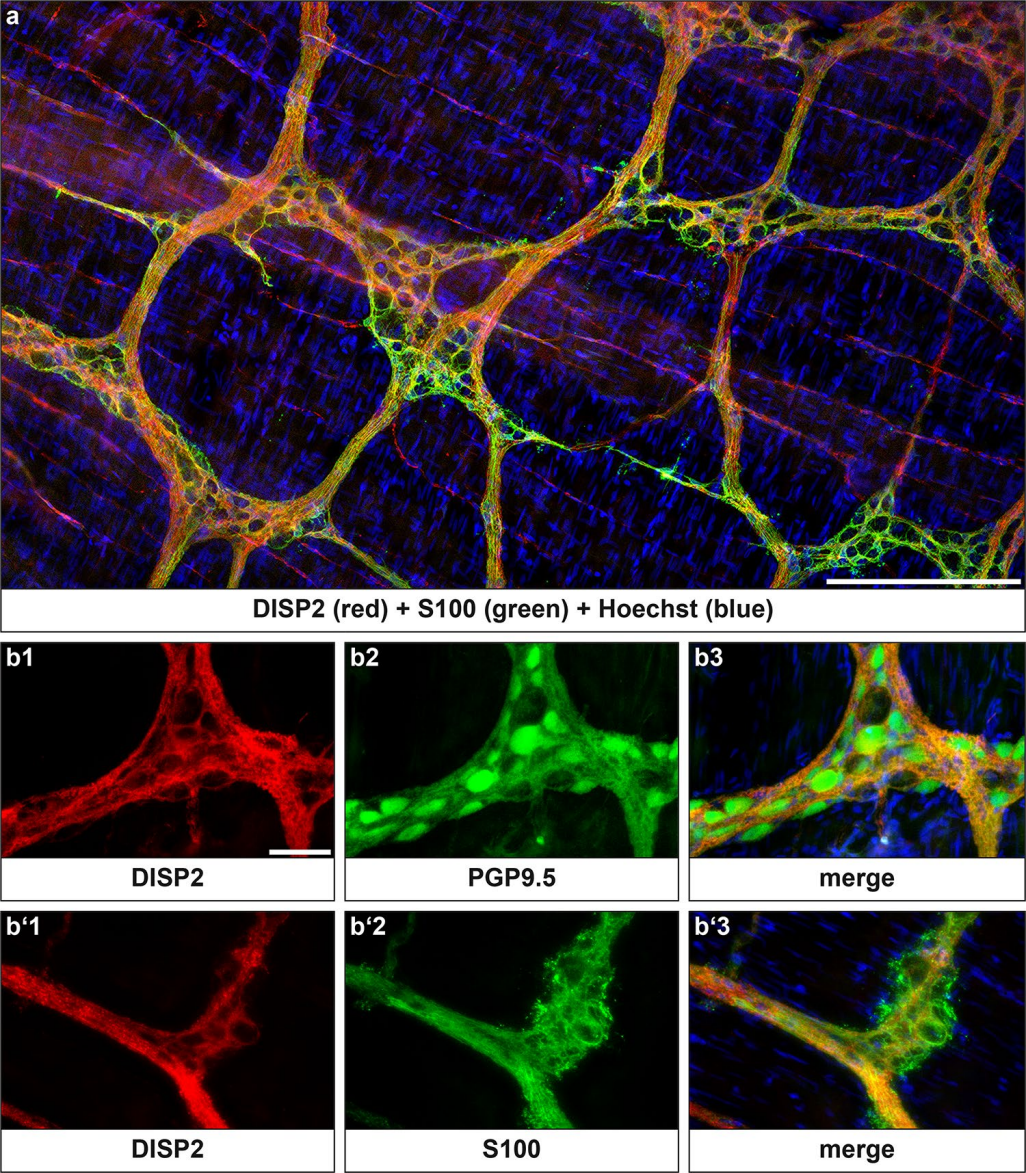
DISP2 localization pattern in adult rat enteric nervous system. Immunohistochemical staining for DISP2 was performed on whole-mount preparation of colonic tissue (**a**). DISP2 (red, **b**1, **b**′1) signals were detected in PGP9.5- (green, **b**2) positive neurons of ganglionic structures that were surrounded by S100-positive glia cell processes (**b**′2). Nuclei were counterstained with Hoechst (blue), and corresponding merge images are shown (**b**3, **b**′3) (scale bar in a = 200 µm, b = 50 µm; *n* = 4)

Using whole-mount preparation of rat colon, DISP2 immunoreactivity was observed in ganglionic structures of the myenteric plexus, as well as in interganglionic nerve fiber strands (Fig. 5a, supplementary Fig. 6). Costaining revealed the protein expression of DISP2 in PGP9.5-positive neurons (Fig. 5b), whereas limited staining was observed in S100-positive glial cells (Fig. 5b′).

**Fig. 6:**
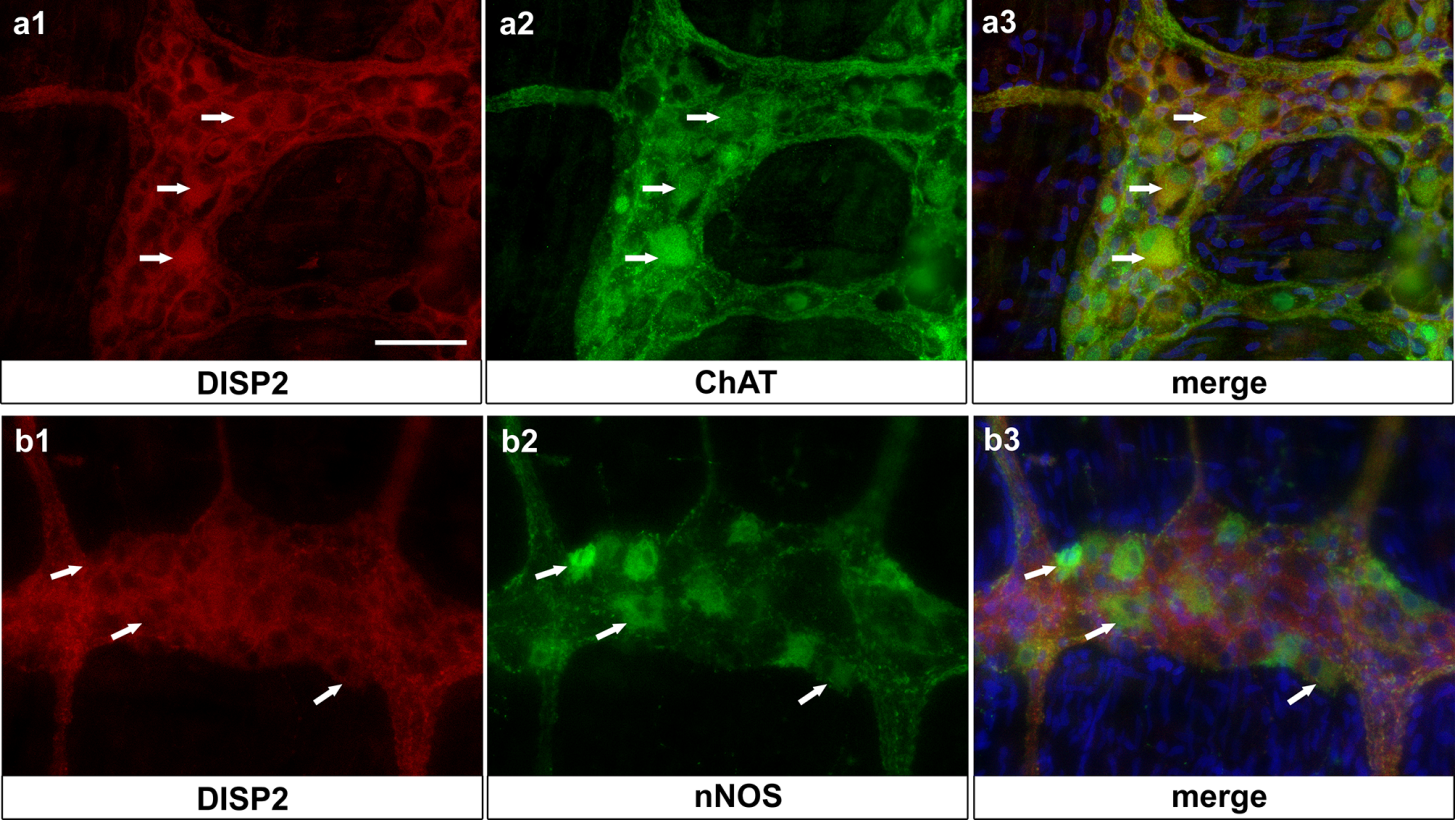
DISP2 localization pattern in cholinergic and nitrergic myenteric neurons of the adult rat enteric nervous system. Immunohistochemical staining for DISP2 (red, **a1**, **b1**) signals was detected in ChAT-positive neurons (green, **a2**, arrows) and nNOS-positive neurons (green, **b2**, arrows) within the myenteric plexus. Nuclei were counterstained with Hoechst (blue) and corresponding merge images are shown (**a3**, **b3**) (scale bar = 50 µm; *n* = 3)

Although intensity of DISP2 immunoreactivity in myenteric neurons was variable, DISP2 was found to colocalized both with ChAT-positive (Fig. 6a) and with nNOS-positive neurons (Fig. 6b).

**Fig. 7:**
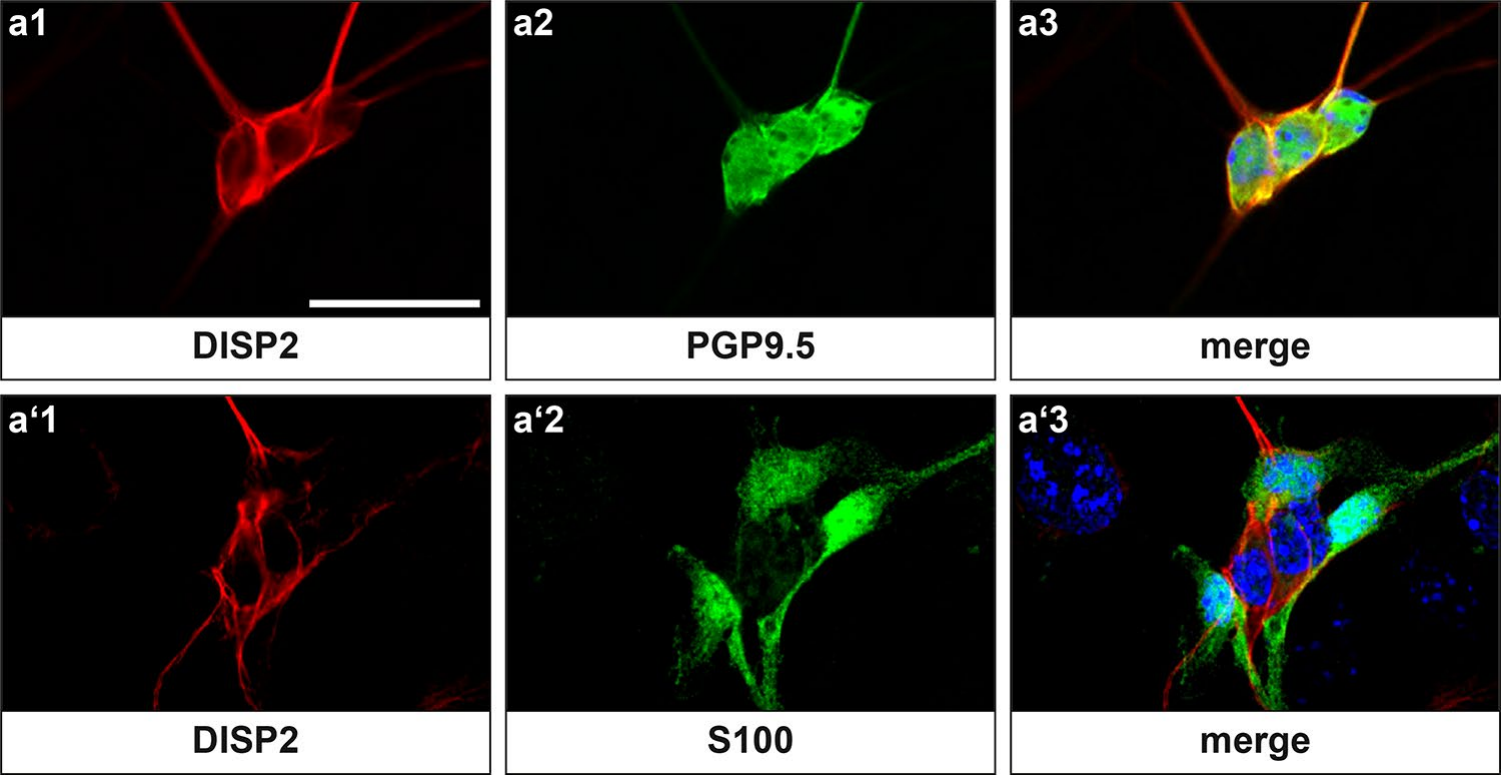
DISP2 localization pattern in primary cultures of murine ENS. Confocal microscopy of primary cultures of murine ENS stained with DISP2 (red, **a**1, **a**′1) and the neuronal marker PGP9.5 (green, **a**2) or the glial marker S100 (green, **a**′2). DISP2 signals were detectable in the neuronal cell somata and processes but only to limited extend in the cell nuclei. Note the presence of S100-positive glial processes surrounding DISP2-positive neuronal processes. Nuclei were counterstained with Hoechst (blue), and corresponding merge images are shown (**a**3, **a**′3) (scale bar = 20 µm; *n* = 4)

In order to confirm the localization of DISP2 in ENS cell populations, DISP2 staining was performed on murine primary cultures of the ENS. Using confocal microscopy, DISP2 staining was observed in PGP9.5-positive neuronal cell somata and in nerve processes (Fig. 7a). These cells were surrounded by S100-positive glial cells, which remained DISP2-negative (Fig. 7a′), confirming the predominant neuronal localization of DISP2 within the ENS. The neuronal nuclei showed only limited, if any, DISP2 immunoreactivity.

## Discussion

Despite accumulating evidence in support of DISP2 in neurocognitive and intestinal neuromuscular diseases, DISP2 localization within nervous tissues has not been reported so far. At transcriptomic level, *DISP2* expression has been proposed to be neuro-specific in the human and mouse brain, based on single-cell analyses of five independent datasets (McKenzie et al. 2018). In accord with the study of McKenzie and coauthors, our study indicates that *DISP2* is expressed in differentiated neurons in the human and murine adult CNS. This localization pattern is also in line with *DISP2* transcriptomic and in situ hybridization profiles available for the mouse from the Allen Brain Atlas (https://mouse.brain-map.org/gene/show/84648, Lein et al. 2007), suggesting that the neuronal specific expression profile of DISP2 is conserved amongst mammals. This assumption is however limited by the fact that we also observed a DISP2 localization in ependymal cells surrounding the ventricles in rat tissues.

Beside in the CNS, our data indicate that *DISP2* is also expressed within the ENS in human and rat adult colonic tissues. Most particularly, DISP2 is mainly localized within neuronal somata and processes in the human and murine ENS. This localization pattern is to a large extent in line with the expression profile obtained from a single-cell transcriptomic dataset for human gastro-intestinal tissues (Fawkner-Corbett et al. 2021). Neuronal diversity in the ENS is highly complex, and novel neuronal subpopulations have been described based on single-cell transcriptomic profiles in the mouse and in human (Drokhlyansky et al. 2020; Fawkner-Corbett et al. 2021; Morarach et al. 2021). Nonetheless, the definition of clear enteric neuronal subtypes based on these different cell-specific transcriptomic profiles is still the subject of ongoing debates (Rosenberg and Rao 2021; Morarach et al. 2021). In our study, DISP2 localization was observed both in ChAT- and nNOS-positive myenteric neurons, which represent two main subtypes of enteric neurons (Morarach et al. 2021). Although DISP2 immunoreactivity was found in most enteric neurons analyzed, both in human and in rat tissues, we also observed variable DISP2 expression levels in the different neuronal subtype populations analyzed. Further work is needed to decipher the relevance of this DISP2 dosage regulation and its correlation with enteric neuronal subpopulations. Besides, we cannot exclude that DISP2 may be expressed in additional, yet not investigated cell populations, including neuroendocrine cells.

In contrary to DISP1, limited data are available regarding the DISP2 functionality. DISP1 knock-out mice die at early stages during embryological development (Burke et al. 1999; Caspary et al. 2002; Kawakami et al. 2002; Ma et al. 2002). Whereas DISP1 appears to perform functions analogous to its *Drosophila* homolog Dispatched, this appears not to be true for DISP2. Indeed, murine DISP1 can compensate for Dispatched deletion in *Drosophila*, whereas DISP2 cannot (Ma et al. 2002). Moreover, in contrary to DISP1, DISP2 fails to induce Hh signaling in zebrafish, suggesting that DISP2 may have lost its ancestral DISP functionality to regulate Hh signaling in the course of evolution (Nakano et al. 2004). DISP3 has been shown to be involved in the maintenance of a progenitor phenotype in neural cells, and the DISP3 expression level influences the neural progenitor cell fate (Zíková et al. 2014; Konířová et al. 2017). Furthermore, the DISP3 expression is regulated by thyroid hormones, and it was suggested that DISP3 might represent a link between thyroid hormone activity and cholesterol metabolism in the brain (Zikova et al. 2009, 2014). Thyroid hormones, in particular 3,5,3′-triiodothyronine, were shown to modulate the proliferation and differentiation of ENS progenitors in vitro (Mohr et al. 2013). Whether DISP2 may be involved in this regulation remains to be determined.

Interestingly, recent GWAS databases indicate that *DISP2* risk loci are associated with a wide variety of pathologies. GWAS have identified *DISP2*-related risk loci for lung cancer, as well as for diverticular disease (Schafmayer et al. 2019; Kachuri et al. 2020). Recently, DISP2 was found downregulated in low grade glioma, as well as in an esophageal cancer cell line (Cai et al. 2015; Liu et al. 2021), suggesting a putative role as a tumor-suppressor protein. Importantly, *DISP2*-associated risk loci have been associated with impaired brain developmental patterns (Cai et al. 2014) and with altered cognitive functions (Davies et al. 2018; Lee et al. 2018; Savage et al. 2018). Of note, *DISP2* associated-loci have also been proposed to be negatively associated with aging in an unpublished report (Sin-Chan et al. 2020, https://doi.org/10.1101/2020.07.19.188789). *DISP2* expression is regulated by miR-214 (Li et al. 2008), a micro-RNA involved in brain development (Irie et al. 2016; Shu et al. 2017), suggesting that DISP2 may similarly be involved in embryonic neuronal tissue development.

To conclude, our results demonstrate that DISP2 is widely localized in neuronal cell populations in the human and murine CNS and ENS. In this regard, it would be of interest to assess for potential alterations of the DISP2 expression in cognitive and neurodegenerative, as well as in intestinal neuromuscular disorders in future studies.

### Supplementary Information

Below is the link to the electronic supplementary material.

Supplementary file1 (PDF 533 KB)

## Data Availability

The raw data that support the findings of this study are available from the corresponding author upon reasonable institutional request.
