# Supplementary material for: Localization Pattern of Dispatched Homolog 2 (DISP2) in the Central and Enteric Nervous System
Source: J Mol Neurosci. 2023 Jun 27;73(7-8):539–48. doi: 10.1007/s12031-023-02129-8 (PMC10517031; doi:10.1007/s12031-023-02129-8)
Supplement: Supplementary file 1 — Supplementary file1 (PDF 533 KB) [file 12031_2023_2129_MOESM1_ESM.pdf]

supplementary data

Supplementary table 1. Demographic data and cause of death of the body donors included in this study.

**Age**	**Sex**	**Cause of death**
88	male	pneumonia
81	male	suspected pulmonary embolism
86	female	myocardial infarction
74	male	aspiration pneumonia
85	male	cardiogenic shock

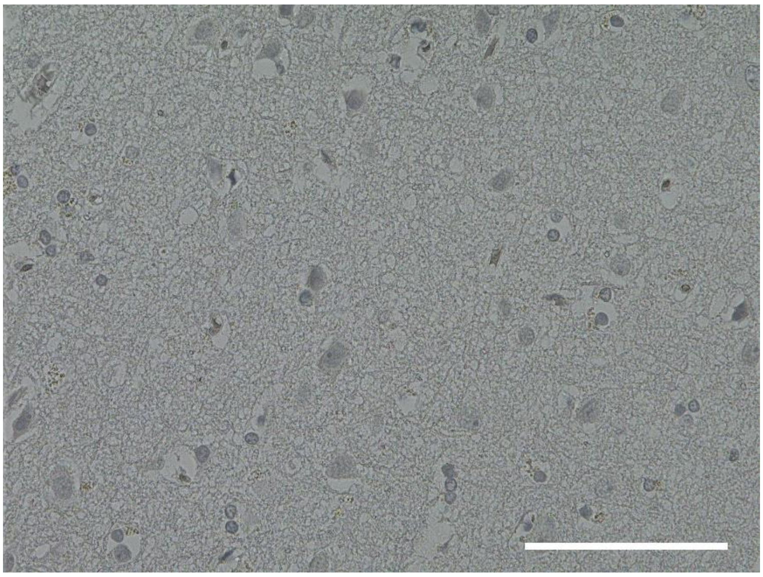

Supplementary figure 1. Negative control for antibody stainings on human brain section. Section of human brain treated with secondary antibodies and the nuclear stain (hematoxylin) as a negative control. The microscope settings were the same as for figure 2. Scale bar = 100 µm

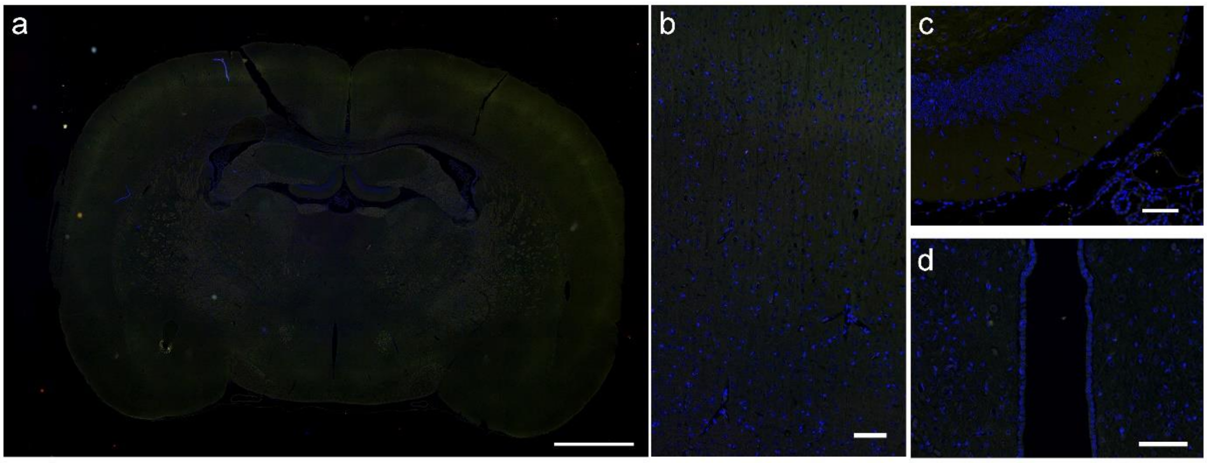

Supplementary figure 2. Negative control for antibody stainings on rat brain section. Section of rat brain were treated with secondary antibodies and the nuclear stain (Hoechst, blue) as a negative control. a) Overview of a whole section, scale bar: 2000 µm. b) Detail of the motor cortex, scale bar 50 µm. c) Detail of the hippocampus, scalebar: 50 µm. d) Detail of ependymal cells, scalebar: 50 µm. The microscope settings were the same as for figure 3.

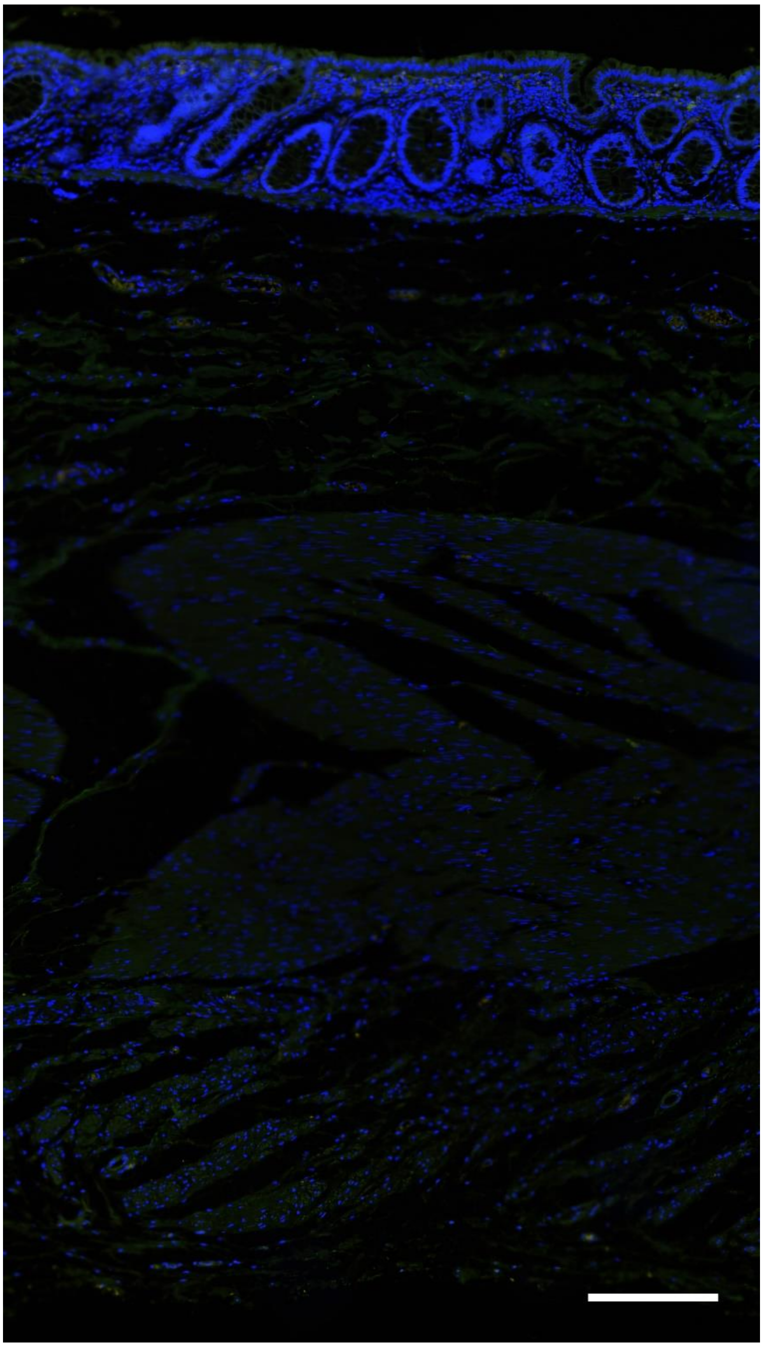

Supplementary figure 3. Negative control for antibody stainings on human colon section. Section of human colon treated with secondary antibodies and the nuclear stain (Hoechst, blue) as a negative control. The microscope settings were the same as for figure 4. Scale bar = 200 µm

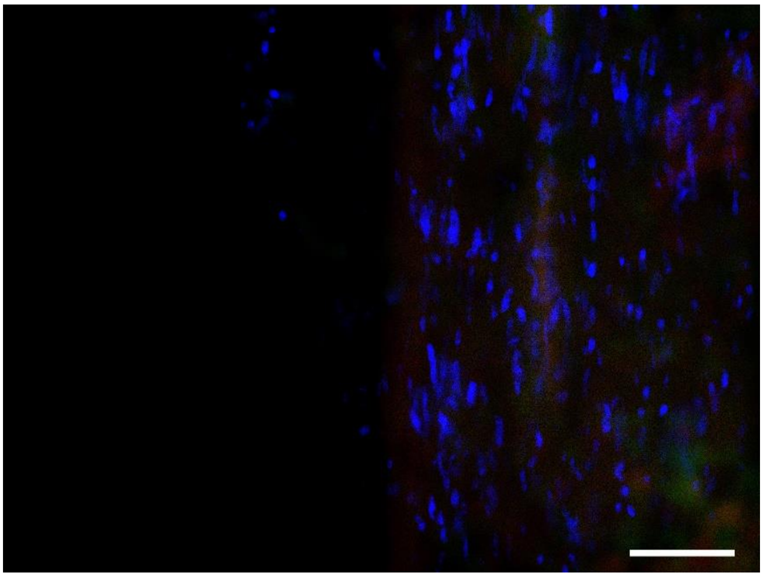

Supplementary figure 4. Negative control for antibody stainings on rat colon section. Section of rat colon treated with secondary antibodies and the nuclear stain (Hoechst, blue) as a negative control. The microscope settings were the same as for figure 5. Scale bar = 50 µm

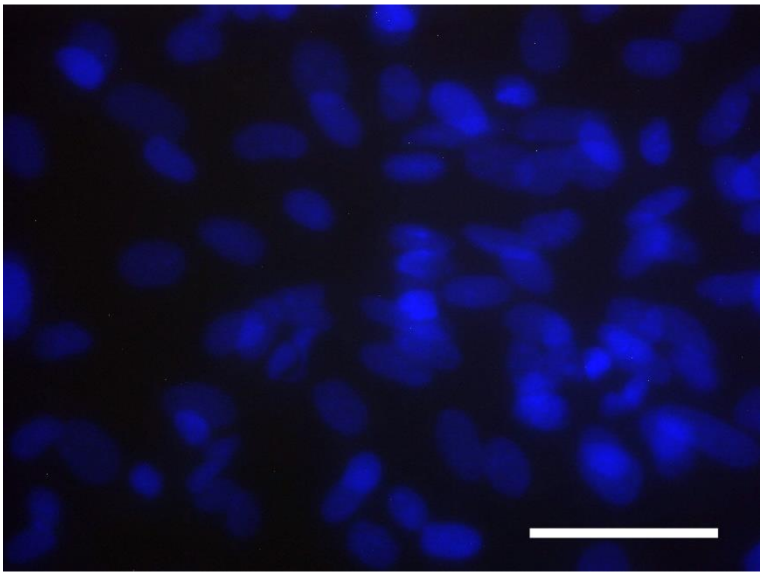

Supplementary figure 5. Negative control for antibody stainings on murine primary culture of ENS. Primary culture of ENS were treated with secondary antibodies and the nuclear stain (Hoechst, blue) as a negative control. The microscope settings were the same as for figure 7. Scale bar = 50 µm

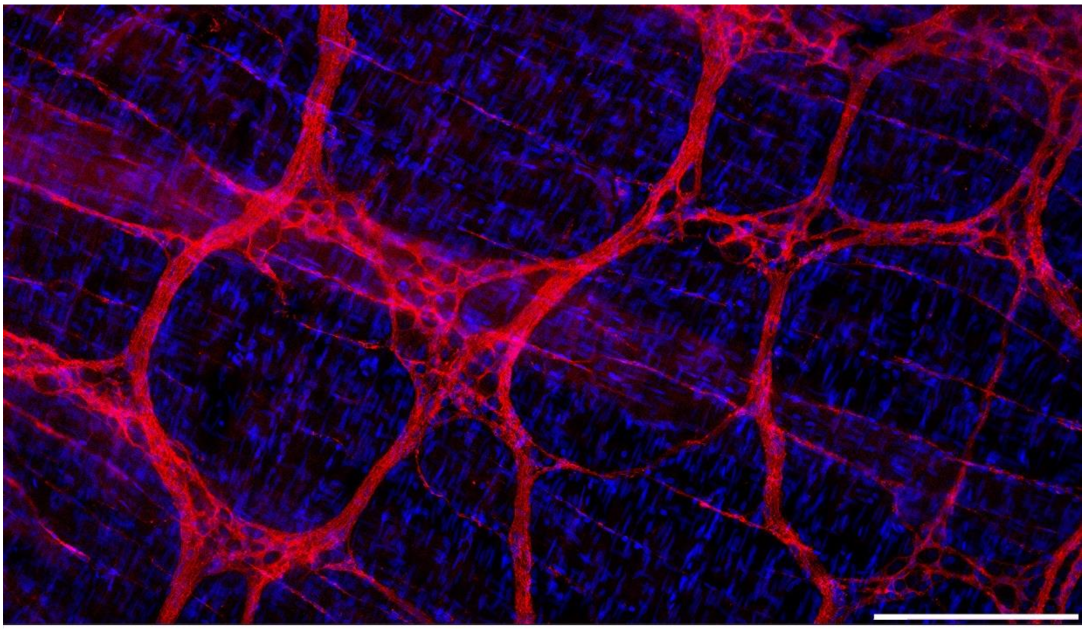

Supplementary figure 6. DISP2 localization pattern in the rat myenteric plexus. Immunohistochemical staining for DISP2 was performed on whole-mount preparation of colonic tissue as presented in figure 5a. DISP2 (red) is shown with cell nuclei (Hoechst, blue). Scale bar = 200 µm
